# Mass Trapping Lepidopteran Pests with Light Traps, with Focus on Tortricid Forest Pests: What If?

**DOI:** 10.3390/insects15040267

**Published:** 2024-04-12

**Authors:** Marc Rhainds

**Affiliations:** Natural Resources Canada, Canadian Forest Service, Atlantic Forestry Centre, P.O. Box 4000, Fredericton, NB E3B 5P7, Canada; marc.rhainds@nrcan-rncan.gc.ca

**Keywords:** artificial light at night, bycatch and insect biodiversity, the economic valuation of protected forests, ‘female removal’

## Abstract

**Simple Summary:**

The attraction of moths to light sources has long been used to reduce the damage of agricultural pests, starting in antiquity with controlled fires in the vicinity of crops. Coinciding with their development in the 18th century, light traps have been used to control pest populations by culling densities of adults lured to death at light sources (light trap-based mass trapping, or *LT_mt_*). Historically, the most extensive large-scale *LT_mt_* trials were conducted in the U.S.A. starting in the 1920s, validating in the process the hypothesis that crop damage can be reduced by removing egg-carrying females from the pest reproductive pool. Light-based mass trapping programs were phased out in the 1970s, coinciding with the implementation of pheromone-based pest management. With the advent of LEDs, solar power sources, and intelligent designs, recent years have seen an uptick of interest in *LT_mt_*, with the majority of contemporary studies conducted in Asia. As a rule, *LT_mt_* trials have been conducted exclusively in agricultural landscapes. A novel approach is proposed here to control epidemic populations of a tortricid forest pest, spruce budworm, in geographically isolated forests of balsam firs at a high risk of intense defoliation and mortality.

**Abstract:**

The management of Lepidopteran pests with light traps (*LTs*) is often achieved by luring adults to death at light sources (light trap-based mass trapping, or *LT_mt_*). Large-scale *LT_mt_* programs against agricultural pests initiated in the late 1920s in the United States were phased out in the 1970s, coinciding with the rise of pheromone-based management research. The interest in *LT_mt_* has surged in recent years with the advent of light emitting diodes, solar power sources, and intelligent design. The first step in implementing *LT_mt_* is to identify a trapping design that maximizes the capture of target pests and minimizes the capture of non-target beneficial insects—with a cautionary note that high captures in *LTs* are not equivalent to the feasibility of mass trapping: the ultimate objective of *LT_mt_* is to protect crop plants from pest damage, not to trap adults. The captures of egg-carrying females in light traps have a greater impact on the efficiency of *LT_mt_* than the captures of males. When *LT_mt_* is defined as a harvesting procedure, the biomass of females in *LTs* may be viewed as the best estimator of the mass trapping yield; biomass proxy has universal application in *LT_mt_* as every living organism can be defined on a per weight basis. While research has largely focused on agricultural pests, an attempt is made here to conceptualize *LT_mt_* as a pest management strategy in forest ecosystems, using spruce budworm as a case study. The mass trapping of female budworms is impossible to achieve in endemic populations due to the large spatial scale of forest landscapes (implying the deployment of a prohibitively large number of LTs); in addition, ovipositing female budworms do not respond to light sources at a low density of conspecifics. The light-based mass trapping of female budworms may provide a realistic management option for geographically isolated forest stands heavily infested with budworms, as a tool to prevent tree mortality. Somehow unexpectedly, however, one factor obscuring the feasibility of *LT_mt_* is as follows: the complex (‘unknowable’) economic valuation of forest stands as opposed to agricultural landscapes.

## 1. Introduction

**Figure 1:**
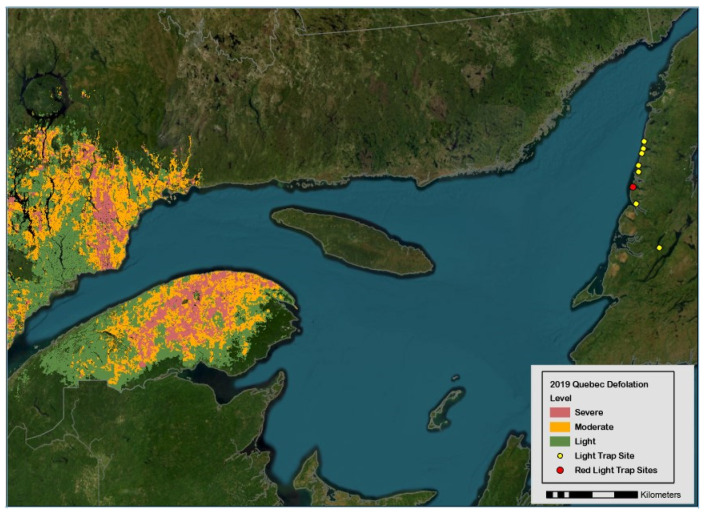
Geographic location of forest sites on west coast of Newfoundland where adult spruce budworms were collected in light traps in 2019 (yellow dots, with red dot representing Sally’s Cove). Defoliated forest stands on the south–north shores of Saint Lawrence River are represented to highlight emigration pressures originating from budworm populations in province of Québec.

The spruce budworm, *Choristoneura fumiferana* Clem. (Lepidoptera: Tortricidae), is a severe epidemic defoliator of conifers [balsam fir, *Abies balsamea*, and spruce trees, *Picea* sp.] in eastern Canada and the northeast states in the U.S.A. [1,2,3]. Concomitant with the large-scale outbreak of budworms in the province of Québec, two massive immigrations of females were detected in light traps (*LTs*) deployed in Sally’s Cove in 2017 and 2018 (red dot in Figure 1), which led to a fortyfold two-year increase in the abundance of overwintering larvae from 0.2 to 8.6 individuals per branch [2] and nearly one million adults in 16 *LTs* in 2019 (Section 3). The initial research question at hand (the recognition of immigration patterns in *LTs*) was defeated by the a posteriori obvious reality that immigration is hard to discern in a high local density of adults. On the other hand, the high numerical abundance (10^6^) in 2019 suggests that budworm populations could in theory be managed by luring females to death with *LTs*—noting that studies explicitly designed to validate mass trapping in agricultural settings rarely attain 10^6^ individuals in *LTs* for any given year, even when hundreds of traps are deployed (Section 2). With some leap of faith, the rationale for mass trapping spruce budworms is inferred numerically.

Unbeknown to many entomologists, the successful control of Lepidopteran agricultural pests is often achieved with *LTs*, either to suppress/eradicate isolated populations (the sustained deployment of *LTs* over multiple generations) [4,5,6] or reduce short-term pest damage by culling populations of reproductive adults (*LT*-based mass trapping per se, or *LT_mt_*). The commonality of *LT_mt_* is highlighted by the large number of field trials testing the approach [7,8,9,10,11], pest management guidelines in multiple crops [12,13,14,15,16,17], and recent deployment of 170 million *LT* units to control agricultural pests in China [18].

The research on *LT_mt_* was pioneered by American entomologists who tested (and often validated) the concept that plant damage caused by insect pests can be mitigated by mass trapping adults in *LTs*: starting in the late 1920s, field experiments carried out over tens of thousands ha led to the capture of several millions adult pests, yielding in the process a trove of quality data (Table 1; see [9] for a detailed review) [6,19,20,21,22,23,24,25,26,27,28,29,30,31,32,33,34,35,36,37,38,39,40,41,42,43,44,45,46,47,48,49,50,51,52,53,54,55,56,57,58,59,60,61,62,63,64,65,66,67,68]. Large-scale *LT_mt_* trials were phased out in the early 1970s, coinciding with the rise of pheromone-based management research. The research output was low worldwide between 1975 and 2010 but steadily increased since; the geographic pole of research has shifted from the U.S.A. to Asia (Table 1).

The main objective of this study is to explore the plausibility (*what if?*) of *LT_mt_* as a management tool for spruce budworm using two steps. (1) Based on a large (but not exhaustive) review of the literature on Lepidoptera, identify the parameters influencing the efficacy of *LT_mt_* (*Ē*) in multiple contexts. (2) Parameterize *Ē* in budworm, in particular a ‘novel’ yield proxy (biomass of trapped females) with universal application.

## 2. Parameters Influencing *E*

Based on the literature review including 51 relevant studies (Table 1), *Ē* was defined relative to the light trapping protocols, demography of pests, and economic impact of *LT_mt_*, as summarized in Table 2. Equations are provided to iterate processes involved in the implementation of *LT_mt_*; equation parameters are described in Table 3.

### 2.1. Light Trapping Protocols

The efficacy of *LT_mt_* relies on trap attributes that simultaneously maximize captures of target pests and minimize captures of beneficial insects. Notwithstanding monetary constraints, *LTs* should be deployed at a density (traps/ha) and height above ground that optimize *Ē*.

#### 2.1.1. Trapping Design

One objective of *LT_mt_* is to identify a specific design (*d_i_*, overall trap configuration including light attributes, power source, funnel, and collecting device) that maximizes the number of target pests in *LTs* (*n_p_*, including ♂ and ♀):

*Ē_i_* = *d_i_* for {*n_p_*} _(max)_ (1)

In principle, trap *i* corresponding to {*n_p_*} _(max)_ is used in large-scale mass trapping trials.

The recent literature is pervaded by a circular assumption as to the identification of the design *i* being near equivalent to the efficacy of *LT_mt_* [19,25,26,29,31,36,37,38,41,69,70,71]. In reality, a high *n_p_* does *not* imply a high *Ē* without accounting for the time-delayed effects of mass trapping on crop damage (Section 2.3).

The attraction/retention of adults in *LTs* often increase with trap size, i.e., the surface of the light source and volume of receptacles where trapped moths are collected [9,31,33,36,43,51,72,73]. Recent trends toward the miniaturization of *LTs* may thus impede the development of *LT_mt_* because small light sources attract relatively few adults and small receptacles rapidly saturate.

The development of novel *LTs* has blossomed in recent years (Table 1) with the advent of light emitting diodes, solar-powered energy sources, and intelligent designs [13,18,26,70,71,73,74,75], leading to a renewed interest in *LT_mt_*. The high rate of innovation in designing *LTs* for insect control is not problematic per se; after all, improvements in trap design have been an intrinsic part of *LT* development since early inception [9]. On the other hand, the high diversity of *LT* designs hampers comparisons between pests/studies hence the need for standardized protocols in plant protection programs [12,76,77,78].

The intractable issue of ever-increasing trap innovation is resolved here with a simple assumption as to *LTs* in different mass trapping studies being near equivalent between studies (‘a trap is a trap’) but obviously not within studies (Section 2.1.4). In any event, *LT* design may be largely irrelevant when captures at the trap level are more strongly impacted by environmental effects than trap attributes [79,80,81].

The mechanisms influencing the response of insects to light sources [circadian rhythms and photoperiod; the physiology of flight-to-light, including moon/abiotic/environmental effects; and interspecific variation in attraction to light attributes] have already been thoroughly reviewed [8,9,10,11,12,13] and deemed outside the scope of this study. In this context, variable captures in *LTs* with different light sources are not accounted for in Table 1 and Table 2, i.e., ‘a trap is a trap’.

#### 2.1.2. Trap Specificity

Because many insect species across all major taxonomic orders are attracted to *LTs* [12,13,14,31,32,35,41,53,73,82,83], mass trapping programs are designed with light sources that predominantly capture key target pests and reduce the attraction of beneficial insects. The problem can be approximated with a redefined optimal trap design *d_j_* associated with the highest ratios of target pests to beneficial insects (*n_p_*/*n_b_*):

*Ē_j_* = *d_j_* for {*n_p_*_/_*n_b_*} _(*max*)_ (2)

Upper boundaries of *Ē_j_* exceeding three orders of magnitude have been reported (1000 individual pests for every beneficial insect) [8,18]. As a general trend among beneficial insects, predatory beetles and lacewings are most often captured in *LTs* [23,29,31,82]. While the notion of ‘friendly’ traps has long been on the mind of applied entomologists [49,53,63], the ratios of pests to beneficial insects were rarely recorded before 1980 (11% of studies) but have since become a mainstay of *LT_mt_* (48% of studies) (Table 2).

For simplicity, the underlying assumption below is that the optimal design *ij* is used in *LT_mt_*.

#### 2.1.3. Density of Light Traps

Because the attraction of adult insects to *LTs* is usually short-range, the performance of *LT_mt_* is strongly dependent on the number of traps per unit area [7,8,9,15,47,49,84,85,86]. Parameter *Ē*, approximated as the cumulative number of pests in all *LTs* (Ʃ*n_p_*, relative to the number of pests in individual traps *n_p_*), is dependent on the density of traps (*T*, on a per ha basis), behavioral/functional response of adults to multiple traps (*k*), and monetary constraints ($) as to the realistic number of traps to be deployed.

*Ē_T_* = *T* × *k* for {Ʃ*n_p_*/$} _(*max*)_ (3)

Coefficient *k* is either 1 when *n_p_* is independent of *T*, <1 when *n_p_* declines with *T*, or >1 when *n_p_* increases with *T*—with a cautionary note that [to the author’s knowledge] rigorous estimates of *k* are not available for any species targeted by *LT_mt_*. In a figurative sense, the deployment of multiple ‘illumination’ traps high above the plant canopy may be viewed as a single large source of light interfering with the reproductive activities of females [20,49,63,84,85] rather than mass trapping per se. In the absence of monetary constraints (the number of trapped pests per $), the optimal resolution of Equation (3) might involve a near infinite number of traps to be deployed.

Indirect approaches to infer *k* include variation in the number of light sources per trap [21,33,78,85] and a variable distance between the traps/number of traps per hectare, in order to evaluate the attraction range of individual traps [15,21,23,30,45,50,86]. The issue is further complexified when males and females exhibit a distinct response to trap density [87].

#### 2.1.4. Position of Traps

The spatial position of *LTs* influences the level of captures. For example, large-scale *LT_mt_* (>10^4^ ha in some instances; Table 1) often includes traps deployed outside treated areas (control traps) to assess *Ē* (Section 2.3).

The height deployment of traps (*h_i_*, ranging between the ground level and tower-mounted traps tens of m high) also influences captures in *LTs*. Overall, nine studies investigated the effects of h_i_ on *LT_mt_* (six before 1980 and three after) (Table 2). In theory, traps should be deployed at a height that maximizes *n_p_* [10,11,15,51]. In practice, however, the magnitude and directionality of height effects are difficult to generalize as they often vary with the plant growth stage [20,23,31,44].

### 2.2. Demography of Target Pests

The feasibility of *LT_mt_* has been investigated in 13 families of Lepidoptera, most notably Noctuidae (12 species), Crambidae (5 species), and Gelechiidae–Geometridae–Tortricidae (3 species each). In total, >30 million adult pests were killed in *LTs* in these studies (including references in [9]), ranging four orders of magnitude (10^2^ to 10^6^ individuals) between studies (Table 1).

#### 2.2.1. Numerical Estimates of Source Populations

From a strict perspective, mass trapping efficiency can be rigorously evaluated if, and only if, an estimate of the local pest abundance is known—so that the proportion of insects successfully mass trapped can be inferred.

*Ω* = Ʃ*n_p_*/*N_p_* (4)

Parameter *Ω*, defined as the ratio between Ʃ*n_p_* and the local density of adults (*N_p_*), is positively correlated with *Ē* [5,8,88,89]. Estimates of *Ω* may reach 98% indoors (apple storage rooms [9]), but precise field values are usually not available in *LT_mt_* studies.

Tools available to approximate *Ω* include estimates of pest abundance before the emergence of adults, modeling seasonal flight patterns in *LTs*, and the mark–release of adults [4,8,9,37,47,48,51,70].

#### 2.2.2. Conspecific Density Effects

It is often assumed that *LT_mt_* is most effective at a low population density, in part based on the following statement: “trapping 80% of a population of 10,000 would have much greater effect than trapping 80% of a population of 1,000,000” [8]. While the argument is intuitively sound, it assumes that captures in *LTs* are proportional to the local population density, i.e., a constant proportion *c* of adults is captured in traps independently of density:

*n_p_* = *N_p_*/*c* for *Ω _┴_ N_p_* (5)

In natural conditions, however, *Ω* is expected to exhibit strong density dependence: attraction to *LTs* reflects both the abundance and dispersal movements of adults, the latter of which often increases with population density [90,91,92]. Assuming positive correlations between *Ω* and *N_p_* (as opposed to a density-independent scenario *Ω _┴_ N_p_*), the original statement above becomes the following: “trapping 80% of a population of 10,000 may have much *lower* effect than trapping >>> 80% of a population of 1,000,000”—implying that *LT_mt_* is, in some circumstances, more effective at controlling epidemic populations than endemic populations (Section 3.2.2).

#### 2.2.3. Sex Ratio

Light traps capture adults of both sexes, and ‘female removal’ is often mentioned as the primary objective of *LT_mt_* [4,8,9,15,28,32,39,45,52,59,60,89]. The mass trapping of males per se is unlikely to suppress female mating success to a near-zero level because a single male can inseminate several female partners during its lifetime [93,94,95].

Captures of egg-carrying females in *LTs* are thus expected to increase the efficacy of *LT_mt_* by factor *S* relative to captures of sperm-carrying males:

*Ē*_♀_ = *Ē*_♂_/*S* (6)

Assuming that *S* is large enough, the abundance of males in *LTs* becomes largely inconsequential. If valid, long-held views that *LT_mt_* prioritizes female targets may imply the recalibration of all equations above based on *n*_♀_ (as opposed to *n_p_* including both sexes).

#### 2.2.4. Average and Residual Fecundity of Females in LTs

The feasibility of *LT_mt_* can be assessed by dissecting females in *LTs* to assess the mating status (the presence/absence of spermatophore) and residual fecundity (eggs in the abdomen of females relative to a full complement at emergence) [22,39,45,52]:

*Ē_egg_* = *n*_♀_ × (*F* − *L*) (7)

For any given species, *F* and *L* represent the average fecundity and proportion of eggs laid by individual females at capture. Virgin/young gravid females with a near-full egg complement are the prime targets of *LT_mt_*; in contrast, captures of old spent females with few eggs in the abdomen are largely irrelevant.

Residual fecundity was commonly reported during the early phase of *LT_mt_* (29% of studies) but not once since 1980 (references in Table 2); these measurements are useful to infer whether mass trapping is a viable option for any pest/crop association. Integrating residual fecundity into Equation (7) remains challenging, however, both logistically (the time-consuming nature of female dissection measurements) and analytically (the intraspecific variation in female body size positively correlates with fecundity in many Lepidopteran species, an effect never accounted for in LT*_mt_* studies).

#### 2.2.5. Body Mass of Females in LTs

The issue above can be simplified in Lepidopteran pest species with non-feeding females by noting that initial weight is set at emergence and monotonically declines over time as eggs are laid [96,97,98]. The cumulative weights of *n* females in LTs (*W*_♀_) ‘recapitulate’ the effects of body size and age on the reproductive condition at capture, e.g., large/young females are heavier than small/old females.

*Ē_W_* = *W*_♀_ (8)

Estimates of mass trapping efficiency are deemed more accurate when expressed in terms of the biomass of females than other proxies (*Ē_W_* > *Ē_egg_* > *Ē*_♀_ > *Ē*_♀,__♂_). The widespread adoption of *Ē_W_* may be constrained by the prevailing mindset focusing on the numerical abundance of adults in *LTs*; only three studies in Table 1 report the weight of adults in *LTs*, none of which differentiated between males and females [23,36,46].

### 2.3. Beneficial Impact of LT_mt_ on Crop Plants

Major pests of economically important crop plants have always been prioritized in *LT_mt_* studies. Before the 1980s, American pioneers of *LT_mt_* focused on Lepidopteran pests of apple, cotton, corn, lettuce, and tobacco. Crops targeted by *LT_mt_* have diversified since then, now including cabbage, eggplant, eucalyptus, legumes, nuts, olive, onion, palm fruits, sugarcane, tea, and tomato (Table 1).

Based on budget itemizations available in some mass trapping studies [18,24,26,39,41,73,99], a simple approach is proposed whereby the incremental yield of crop plants in plots treated with *LTs* (*Y = Y_LTmt_* – *Y_control_*, in $) is assumed to be dependent on a single factor:

*Y* ≈ $/*W*_♀_ (9)

In principle, *LT_mt_* can be recommended when the cost ($/*W*_♀_) is low.

Parameters related to *Y* were assessed in the majority of studies in Table 2 (>60% before and after 1980), including the density of eggs/larvae in offspring generations, proportion of damaged plants, yield estimates, and reduction in the number of insecticide applications (references in Table 2). The efficacy of *LT_mt_* is often distance-dependent: crop damage is the highest further away from mass trapping plots [21,24,26,41,59,63]. Some mass trapping studies did not record adult abundance, focusing instead on measurable consequences of *LT_mt_* on *Y* [21,24,53]. The numbers of mass-trapped females do *not* need to be recorded to assess the benefit of mass trapping, provided that the strong attraction of target pests to traps deployed in *LT_mt_* is demonstrated.

Somehow surprisingly, the comparison of plant damage in the control versus light-treated plots suggests near-universal positive effects of *LT_mt_* on *Y* (*Y_LTmt_* > *Y_control_*) (references in Table 2; see also [8,9,39]) with two caveats. (1) Publication bias may lead to an artificially high rate of *LT_mt_* success if studies failing to document reduced plant damage are unlikely to be published or to report said measurements. (2) The monetary benefit associated with the yield increment is often dwarfed by the cost of mass trapping [8,20,21,60], in which case *LT_mt_* does *not* provide effective pest control.

## 3. Spruce Budworms at Light Traps: Experimental Data

**Figure 2:**
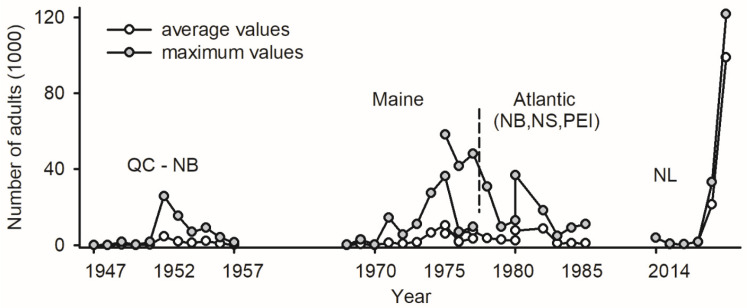
Abundance of adult spruce budworms in light traps in Canadian provinces of Québec and New Brunswick between 1947 and 1957 [101], in state of Maine between 1968 and 1977 [102,103], in Atlantic Canada between 1976 and 1986 (provinces of New Brunswick, Nova Scotia, and Prince Edward Island) [104], and on west coast of Newfoundland (Sally’s Cove) between 2014 and 2019 [2].

The deployment of *LTs* as a monitoring tool for budworms nearly coincided with the first report of a positive phototactic reaction in adults [100]: traps were deployed in multiple locations in Québec and New Brunswick between 1945 and 1957 (4–26 sites each year) to record the abundance of unsexed specimens [101]. The data for each site/year included short time series of abundance with at least ten consecutive days; in total, >250,000 adult budworms were captured in LTs (Figure 2). Due to the age of the data, the attributes of *LTs* are unknown.

The monitoring of adult budworms with *LTs* was implemented in Maine (1968–1977) and Atlantic Canada (1976–1986), with 6 to 19 sites sampled each year for the two jurisdictions (the trap design, location of sites, and raw data in [102,103,104]). The abundance of unsexed budworms in *LTs* was recorded every day for the entire flight season in each site/year, providing reference material for future phenology studies. In total, >350,000 budworms were captured in Maine and >750,000 in Atlantic Canada (Figure 2).

Light trapping conducted at Sally’s Cove on the west coast of Newfoundland (NL) between 2014 and 2019 yielded > 1,200,000 sexed budworms, the majority of which (>80%) occurred in 2019 (Figure 2). A historical increase in the abundance of budworms in *LTs* on a per trap basis over 70 years (maximal values of 26,000 budworms in 1952, 36,000 in Maine in 1975, 58,000 in Atlantic Canada in 1976, and 120,000 in NL in 2019) likely reflects increasingly efficient trap designs (Figure 2). To the author’s knowledge, the number of adults in *LTs* represents the only proxy of budworm abundance available for the last three outbreaks.

### 3.1. Light Trapping Protocols

Sixteen stainless steel vane *LTs* (Leptraps, Georgetown, KY, USA) with a 15 W white neon tube as a light source and powered by marine batteries were used to capture adult budworms in forest stands dominated by balsam fir at eight locations in central Western Newfoundland in 2019 (Figure 1, Annex I, Supplementary Materials; Ref. [2,96,105]). A summary of trap captures in the 16 *LTs* in 2019 is provided in Table 4.

**Figure 3:**
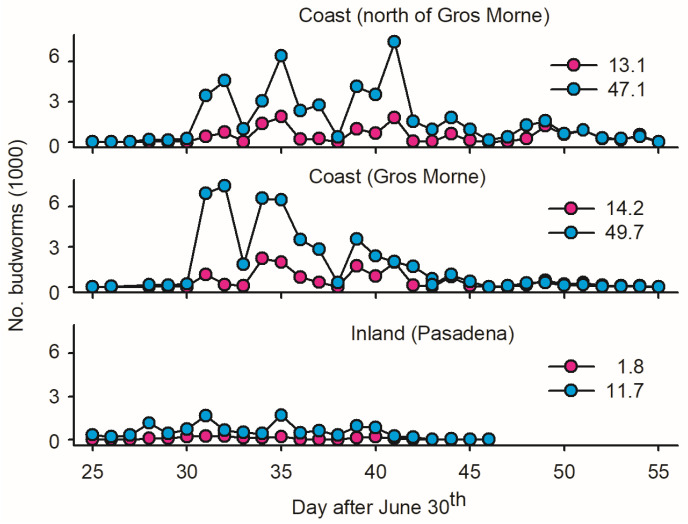
Average number of male and female spruce budworms (pink and blue dots, respectively, averaged on per light trap basis) at three locations on west coast of Newfoundland: (1) one inland light trap in Pasadena; (2) twelve light traps deployed in coastal forests within Gros Morne National Park; and (3) five light traps deployed in coastal forest north of Gros Morne. Location of sites as depicted in Figure 1.

One ‘inland trap’ in Pasadena, 40 km from the coast, served as the control to evaluate the geographic variation in mass trapping feasibility. The data suggest a low *Ē* inland in terms of females per trap (eight-times-lower estimates than on the coast; Figure 3) and a high incidence of non-target insects (Section 2.1.2).

#### 3.1.1. Trapping Design

The traps used in this study performed well relative to *LTs* deployed in previous outbreaks (Figure 2). Of all trap attributes *i* corresponding to the design *d_i_* used here (light source *l_i_*, funnel *f_i_*, volume *v_i_*; Table 3), the latter (the small volume of the collecting bucket below the funnel; *v* = 10.5 *l*) may have constrained the captures of budworms to the largest extent: *LTs* appeared to saturate when the abundance/biomass of budworms exceeded 16,000 individuals/160 g on a fresh weight basis (Annex I, Supplementary Materials). The effect of *v* on the efficiency of *LT_mt_* can be assessed as follows:

*Ē_i_* = *v_i_* for {*n_p_*} _(max)_ (10)

Sampling all traps every day, as conducted in 2019, would be prohibitively costly in an operational *LT_mt_* program. In particular, the problem of trap saturation becomes more stringent if traps are emptied every n^th^ day as opposed to every day. Trap capacity *v_i_* may be increased by retrofitting *LT* on 200 *l* barrel drums (Figure 2 in [39]).

#### 3.1.2. Trap Specificity: Bygone Ghost of Bycatch

Resolving the issue of bycatch (the sum of non-target insects captured in *LTs*) is critical to implement *LT_mt_*: separating targets from non-targets is labor-intensive and hazardous (exposure to insect allergens and decomposition volatiles, mostly if *LTs* are left uncollected for several days).

Surprisingly, the *LT_mt_* literature rarely mentions the term ‘bycatch’. Issues of bycatch may have become irrelevant with the development of ‘friendly’ traps or with the detailed records of abundance for multiple taxonomic groups/species in *LTs* (references in Table 2); without explicit statements by the authors, it is hard to conclude.

Discarding bycatch in *LTs*, either physically or conceptually, is unfortunate. The parameterization of the *LT_mt_* efficiency in terms of biomass provides a simple direct comparison of target (*p*) versus non-target insects (*←p*):

*Ē_j_* = *d_j_* for {*w_p_*_/_*w_¬p_* } _(*max*)_ (11)

The ratio of target/non-target biomass is highly relevant to *LT_mt_* in budworms, with broadly similar bycatch constraints to the fishing industry [106,107,108].

**Figure 4:**
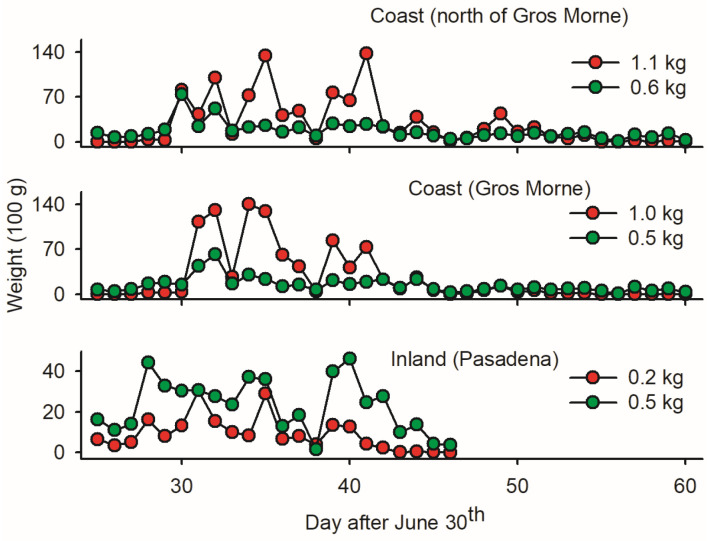
Average cumulative fresh biomass of adult spruce budworms and bycatch (red and green dots, respectively, averaged on per light trap basis) at three locations on west coast of Newfoundland: (1) one inland light trap in Pasadena; (2) twelve light traps deployed in coastal forests within Gros Morne National Park; and (3) five light traps deployed in coastal forest north of Gros Morne. Location of sites as depicted in Figure 1.

The low ratio of budworm biomass relative to non-targets in *LTs* in Pasadena (0.2 versus 0.5 kg; Figure 4) implies that inland mass trapping is not sound due to the substantial removal of beneficial insects. In coastal sites, in contrast, the mass of budworms was two times larger than the mass of non-targets (Figure 4)—suggesting a ‘limited’ non-target cost of *LT_mt_* in budworms. Based on *w_p_/w_b_* ratios, the first ten days of August provided optimal timing for mass trapping budworms.

#### 3.1.3. Density of Light Traps

The estimates of *k* are expected to fluctuate non-linearly with *T*, i.e., differential responses of adults exposed to 1–5 traps/ha versus 5 to 25 traps/ha. The problem is illustrated using relationships between the range of the attraction (*r*, in m) of omnidirectional *LTs* (360° light irradiation) and the number of resident adults available for trapping (*N_p_*):

*N_p_* = Π (*r*/2) ^2^ (12)

The range of attraction to *LTs* in Lepidoptera generally varies between a few meters and about 150 m [109,110,111], although estimates as high as 3 km have been reported [47].

**Figure 5:**
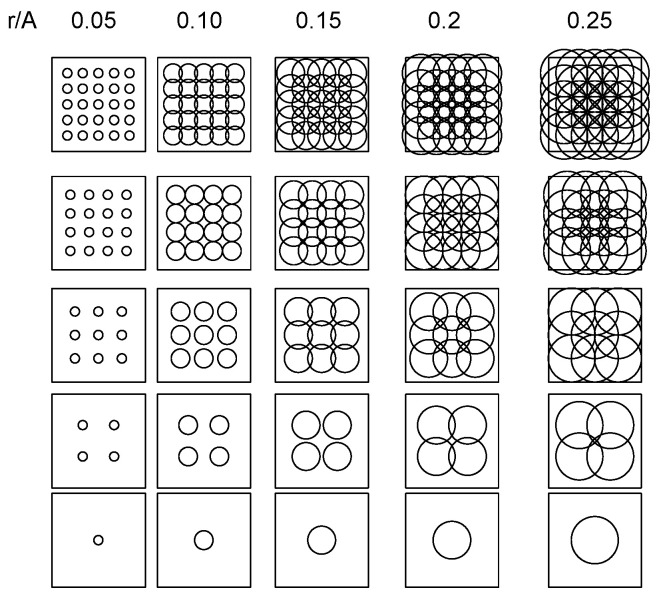
Relationships between surface area illuminated by variable number of light traps (*T* = 1, 4, 9, 16, and 25) deployed in evenly spaced grids within square plot with dimension A^2^, as resolved for different attraction ranges of light sources (*r* in m, ranging between *r*/*A* = 0.05 and 0.25).

For any grid of *T* traps evenly deployed within a square area *A^2^* (*T =* 1, 4, 9, 16, and 25 traps in the example here), *n_p_* is expected to strongly increase with *T* when *r* <<< *A* (left plots in Figure 5); as *r* increases relative to *A*, *np* becomes independent of *T* due to the overlapping attraction between traps (right plots in Figure 5).

#### 3.1.4. Position of Traps

The vertical distribution of flight in insects usually ranges between 0.2 and 3.8 m above ground [112]. Light traps used to attract budworms are either deployed in the upper canopy of host trees > 5 m high [113,114] or (as in this study) on low branches 2.5 m above ground [2,105,115]. Oftentimes, the vertical position of traps is not reported [101,102,103,104], and no direct comparison of budworm abundance in relation to the height of *LTs* is available.

### 3.2. Demography of Spruce Budworms in Light Traps

**Figure 6:**
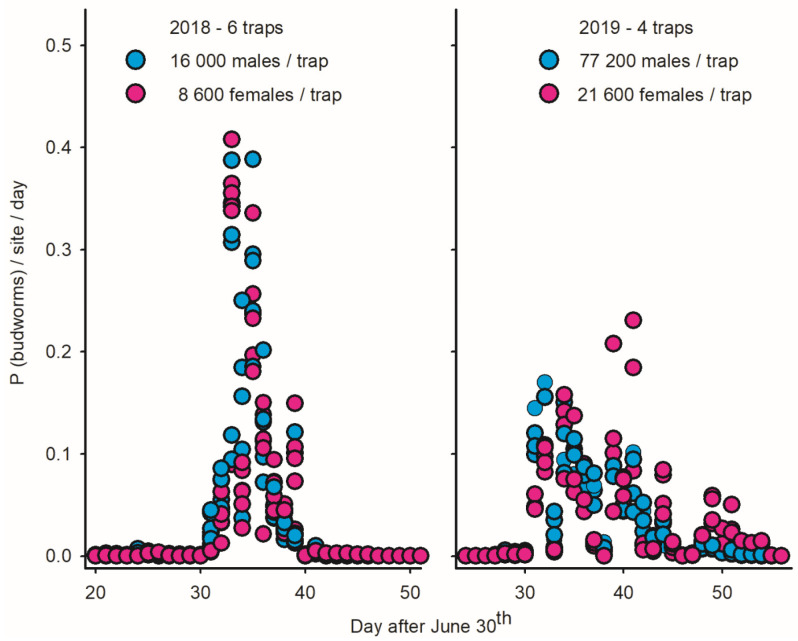
Proportion of male/female spruce budworms captured on daily basis in light traps deployed in Sally’s Cove on west coast of Newfoundland in 2018 (N = 6 traps) and 2019 (N = 4 traps). (**Left**) Immigration in SC in late July–early August 2018 is inferred based on strong synchrony of captures in light traps for both males and females [2]. (**Right**) Strong variation in phenology between traps/sexes in 2019 suggests that immigration was indiscernible due to high population density of resident adult budworms.

Because immigrations are pulsed in time and include both males and females, they are characterized by specific patterns in *LTs*: (1) a large increase in the abundance of males and females from one night to the next; (2) high numbers of budworms following immigration due to the post-migration longevity of adults; (3) strong phenological synchrony among traps when the inter-trap distance is small relative to the spatial scale of the immigration event; and (4) a similar seasonal timing of flight for males and females when immigrants >>> residents (as opposed to protandrous flight in closed populations with residents >>> immigrants) [2]. The signature traits of immigration in *LTs* are illustrated for Sally’s Cove in 2018 (left plot in Figure 6).

While the abundance of budworms in SC increased fourfold between 2018 and 2019 (25,000 to 100,000 individuals per trap), the flight patterns observed in *LTs* in SC in 2019 (as well as other coastal sites) were inconsistent with the scenarios of immigration (right plot in Figure 6), in particular a lack of phenological synchrony between traps and significant protandry in 15 of the 16 *LTs* (right plot of Figure 6; Table 4). These observations do *not* imply that immigration did not take place in 2019 but rather that they were indiscernible due to the high local density of residents.

#### 3.2.1. Numerical Estimates of Source Populations

The intensity of immigration in budworms (the number of females per ha) can reach 10^5^ [116] and possibly >10^7^ when accounting for three-dimensional swarms of budworms in the troposphere (Figure 5 in [117]).

In closed populations with limited immigration, estimates of *N_p_* range between 10^3^ and 10^5^ adults per ha in endemic and epidemic populations [118]. Considering that budworms emerge over a 2–3-week period in July–August [2,96,105], the number of budworms available for trapping on any given night is assumed to be < 10^4^ adults/ha when computing the efficiency of *LT_mt_* (*Ω*):

*Ω* = *n_p_*/10^4^ ha^−1^ (13)

By definition, values of *n_p_* exceeding 10^4^ adults/ha (corresponding to *Ω* > 1) are assumed de facto indicative of immigration.

**Figure 7:**
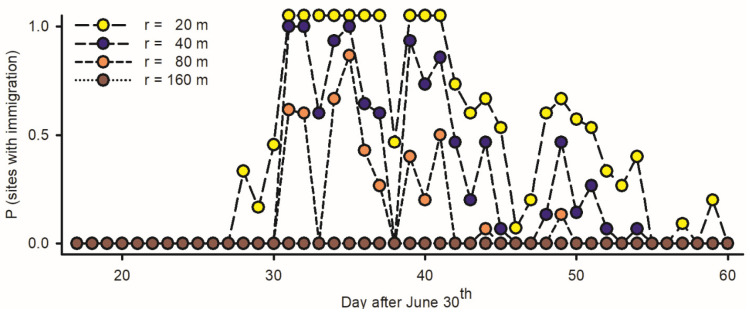
Estimated rate of immigration among adult spruce budworms at fifteen light traps deployed in west coast of Newfoundland in 2019, based on number of specimens at individual traps (*n_p_*) relative to maximal density of inflight resident moths on any given night (N_p_ = 10^4^ adults per ha) [incidence of immigration (*II*) for any given night at each light trap defined as P (*n_p_* > *N_p_*)]. Because captures of budworms are dependent on range of attraction of light traps (Equation (6): *r*, in m), rates of immigration were computed with different values of *r* each corresponding to specific number of resident adults available for trapping each night.

An inference as to the incidence/non-incidence of immigration is dependent on the range of the attraction of *LTs* (*r*, in m): the numbers of budworms available for light trapping per night (Equation (6)) range between *N_p_* = 315 adults/ha if *r* = 20 m, *N_p_* = 1258 if *r* = 40 m, *N_p_* = 5034 if *r* = 80 m, and *N_p_* = 22,650 if *r* = 160 m. The incidence of immigration in different traps (the number of nights with *n_p_* > *N_p_*) is inversely proportional to the range of attraction: (1) if *r* is low (20 m), immigrations are assumed to have taken place in all traps during ten nights between 31 July and 10 August and in most traps thereafter; (2) if *r* is intermediate (40–80 m), immigrations took place at all traps during a few nights and at some traps most nights; and (3) if *r* is large (160 m), immigration may not have taken place at all during the interval of study (Figure 7). Observations at night suggest *r* > 20 m in budworms.

#### 3.2.2. Conspecific Density Effects

The sex-specific density responses of adult budworms in *LTs* [119] can be summarized as follows: (1) captures of males are more or less proportional to the local population density with constant proportion *c* captured in traps (linear response) and (2) females are rarely captured in LTs when the density is low, as approximated with exponential parameter *e*:

*n*_♂_ = *N*_♂_/*c*, with *Ω _┴_ N*_♂_ (14)

*n*_♀_= *N_♀_ ^e^*, with *Ω* ^┬^ *N*_♀_ (15)

While neither *c* nor *e* is known, Equations (14) and (15) are biologically sound when contrasting high (density-independent) dispersal activity among promiscuous mate-seeking males resulting in high captures in *LTs* (*Ω _┴_ N*_♂_) relative to coy egg-laying females not responding to *LTs* unless foliage resources become depleted (*Ω* ^┬^ *N*_♀_) [90,91,92,120,121,122].

#### 3.2.3. Sex Ratio

The sex ratio in resident budworm populations is approximately 1:1 as determined by field collections of pupae or adults sampled on host trees (either with sweep nets or by fogging trees with insecticides) [113,119,123]. As is generally the rule in moths [124,125,126], the sex ratios of budworms in *LTs* are most often male-biased [2,96,105,117,119].

The early emergence of males relative to females (protandry) is ubiquitous in moths [127,128] including budworms: protandrous flight patterns were detected at 15 of the 16 *LTs*, with males flying 2.3 days before females on average (Table 3).

Considering that the vast majority of females in feral populations of budworm are mated, as referenced in [129], parameter *S* in Equation (6) is assumed high enough that only the capture of females influences *Ē*. In principle, early season trapping consisting of mostly males does not contribute to *LT_mt_*.

#### 3.2.4. Average and Residual Fecundity of Female Spruce Budworms in LTs

Female budworms attract males for mating with sex pheromones released shortly after emergence and thereafter lay eggs in a batch on the foliage of host trees; fecundity averages 200 eggs per female [118,123,130,131]. Female budworms exhibit an unusual reproductive strategy (inter-reproductive migrations) with sequential sedentary–dispersive phases [113,117,132]. I. Young gravid females are in principle incapable to sustain flight/migrate due to a heavy abdomen full of eggs and thus assumed not available for light trapping. II. After having laid approximately 50% of their eggs, partly spent females readily disperse and fly to *LTs*, especially so at a high density of conspecifics [133,134,135].

The prime targets of *LT_mt_* are young females with a near-full egg complement, as opposed to nearly spent females with <50% eggs remaining in the abdomen. Inasmuch, a physical impossibility to capture near-gravid female budworms in *LTs* (immutable physiological flight constraint related to wing load) would be extremely detrimental to *LT_mt_*. Virgin females are observed in *LTs* in some conditions, however, as in populations with extreme female-biased sex ratios following the extermination of early season males with DDT [136]. Egg dumping, a common behavior in virgin female budworms, may have evolved as a strategy to facilitate flight take-off [137,138]. In addition, a dispersing morph of females in depleted forest stands (large wings and low body mass) readily fly to LTs after having laid ca. 25% of their egg complement [133,139,140].

**Figure 8:**
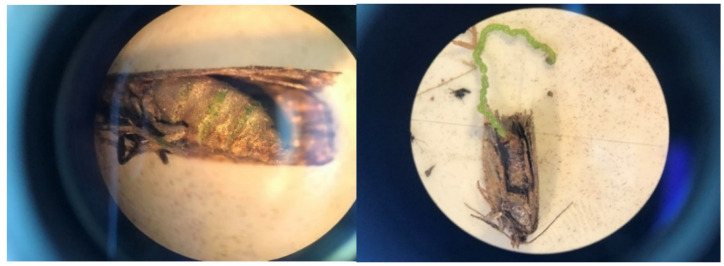
Gravid female spruce budworm captured in light trap on west coast of Newfoundland in early August 2019. Left: plump abdomen filled with green eggs visible through integument. Right: eggs laid by gravid females while being processed for demographic assessments.

Gravid females may be more common in *LTs* than generally assumed due to a methodological flaw when processing adults. During a 5 to 7 d interval in early August 2019, 2 to 5% of female budworms in many *LTs* were gravid (a plump abdomen filled with green eggs often visible through the integument; Figure 8). No formal records were made at the time; instead, *LT* samples were stored in paper bags labeled by sites/dates and frozen for 48 h for subsequent data assessments. After 48 h freezing exposure, unfortunately, the ‘obvious’ plump abdomen of fresh (unfrozen) gravid females had become unrecognizable. It is disconcerting that a procedure routinely used over the years (freezing specimens before processing) leads to systematically biased observations.

#### 3.2.5. Body Mass of Females Spruce Budworms in Light Traps

Biomass is universal in nature: all living organisms can be defined (and compared) on a per weight basis. When *LT_mt_* is viewed through the lens of harvesting [99,141], the biomass of females in *LTs* (*w*_♀_) is hypothesized, not only to provide the best approximation of *Ē* but also to have universal application for any other pest targeted by *LT_mt_*.

The widespread adoption of biomass parameterization may be confronted with the objective of *LT_mt_* generally conceived as to maximize the number of adults in *LTs*, sometimes with a focus on females. When pests are strictly defined on a numerical basis, however, adults become dimensionless points with no physical size.

Female budworms captured in *LTs* are heavier than males: 420 of 424 (99.1%) daily *LT* samples in 2019 including at least two adults of each sex yielded *w*_♀_/*n*_♀_ ≥ *w*_♂_/*n*_♂_. Assuming that only captures of females in LTs contribute to *Ē*, the intraspecific measure of *LT_mt_* performance can be approximated with either the numerical or biomass sex ratio:

*Ē_(n)_* = *n*_♀_/(*n*_♂_ + *n*_♀_) (16)

*Ē_(w)_* = *w*_♀_/(*w*_♂_ + *w*_♀_) (17)

Consistent with the higher weight of females than males, the estimates *Ē_(w)_* are 1.23 times higher than *Ē_(n)_*, i.e., the number of females represented 21.8% of budworms in *LTs* relative to 26.7% female biomass (Table 5).

### 3.3. Management of Budworm Infestations in Gros Morne National Park with LT_mt_?

The management objective of *LT_mt_* in budworms is *not* to prevent outbreaks, first as it is impractical due to the vast spatial scale of forest landscapes and second due to females ignoring LTs at low conspecific density. Instead, *LT_mt_* aims at mass trapping females in geographically isolated outbreaks to prevent tree mortality. Starting in 2021, on the west coast of NL, populations of late instar budworms were treated with *Bacillus thurigiensis kurstaki*; Btk treatments were not authorized at Gros Morne National Park to avoid the mortality of native caterpillars.

With hindsight, sites within Gros Morne (Rocky Harbor: RH; Sally’s Cove: SC; Cow Head: CH) should have been prioritized for research over sites north of the park (Table 3). The potential of *LT_mt_* is likely the highest in SC than other sites in the park for three reasons: the (1) small area and geographical isolation of forest stands surrounded by wetland; (2) high captures of budworms and target/non-target ratios relative to other sites (Table 3); and (3) proximity to the coast in SC (<100 m, relative to >1 km in RH–CH) imply an enhanced likelihood to mass trap immigrant female budworms before they oviposit in the park.

The quantitative pest management objectives of *LT_mt_* are (in ascending order of importance) to reduce larval density in offspring generations, limit defoliation, and prevent the mortality of balsam fir. The biodiversity loss associated with bycatch in *LTs* is not accounted for here, with the caveat that such loss may be deemed unacceptable to park management or potentially worse than the Btk-induced mortality of native caterpillars.

In the worst-case scenario, severe budworm defoliation and the associated massive mortality of balsam fir, combined with a limited prospect for natural regeneration due to high moose density and the consequent browsing of seedling trees [142,143], imply that the coastal forest stand in SC is at a high risk (Ȓ = 1) of transiting to a wetland ‘ghost forest’ with predominantly dead trees [144,145]. In the best-case scenario, the forest stand in SC may be at low risk (Ȓ ≈ 0)—noting that centuries-old coastal balsam firs have so far survived a large number of budworm immigrations.

In theory, the objective of *LT_mt_* in SC can be quantified by computing the yield of mass trapping (ca. 4 kg fresh female biomass/$ 10,000), economic valuation of the forest stand in SC (*V*), and risk of conversion to a swamp ghost forest (Ȓ):

*Ē_SC_*: *ƒ* (*w*_♀_/$, V, Ȓ) (18)

The plausibility of *LT_mt_* (defined as a tool to reduce defoliation/tree mortality caused by budworms) is ultimately determined by the economic valuation of coastal firs in SC relative to the wetland (including ecosystem services, biodiversity value, touristic attributes, etc.). Somehow unexpectedly, a complex monetary assessment of forest value [146,147,148] seems to be the major factor obscuring the application of *LT_mt_* in forestry versus agriculture.

## 4. Conclusions

With the future assumed here to be unknowable, the full-fledged potential of *LT_mt_* against Lepidopteran agricultural pests cannot be ascertained. That said, cautionary optimism is warranted when considering the contemporary uptick in research interest, low cost of light traps, and environmentally friendly nature of *LT_mt_* relative to insecticide applications. The outlook of *LT_mt_* against forest defoliators is more uncertain than in agriculture because the approach is explored here for the first time in spruce budworms.

As a general conclusion, six maxims are proposed as major take-home messages for future light-based mass trapping studies:

(1) The ultimate objective of mass trapping is to reduce crop damage, *not* to increase captures in traps per se.
(2) Imperative for standardized trapping protocols in pest management programs, the hyper-inflationary rate of trap innovation has become counter-productive.
(3) The weight of the bycatch in traps (non-pests) provides a simple proxy of the biodiversity loss in traps.
(4) In general, females captured in traps matter, males do not.
(5) Gravid females with a near-full egg complement are the primary targets of mass trapping.
(6) The weight of female pests at traps provides a universal proxy to quantify the yield of mass trapping, i.e., biomass is universal in nature.

## Figures and Tables

**Table 1:** Summary of field studies evaluating feasibility of light trap-based mass trapping in Lepidoptera, as determined by literature search in Google Scholar using combinations of following keywords (V: and/or): “light trap” V; “mass trap” V control, V pest, V Lepidoptera. Values of multi-year studies are averaged on per year basis: surface area, in ha; number of traps, or T; and number of adults captured, or *n_p_*.

Code	Family	Species	Year	Location	Crop	ha	T	*n_p_*	Refs.
1.1	Cossidae	*Zeuzera pyrina*	2003–2005	Egypt	Olive	2 × 10^0^	1 × 10^1^	3 × 10^2^	[6]
1.2			2014–2015	Iran	Walnut	1 × 10^1^	1 × 10^1^	4 × 10^2^	[19]
2.1.1	Crambidae	*Ostria nubilalis*	1938	Indiana	Corn	3 × 10^0^	2 × 10^1^	6 × 10^3^	[20]
2.1.2			1958–1962	Indiana	Corn	1 × 10^0^	1 × 10^1^	-	[21]
2.2		*Diatraea saccharalis*	1970	Louisiana	Sugarcane	1 × 10^1^	1 × 10^1^	7 × 10^2^	[22]
2.3		*Coniesta ignefusalis*	1993	Niger	Millet	3 × 10^0^	6 × 10^1^	6 × 10^3^	[23]
2.4		*Crocidolomia binotalis*	2018	Indonesia	Cabbage	1 × 10^−2^	6 × 10^0^	1 × 10^3^	[24]
2.5		*Leucinodes orbonalis*	2018	Pakistan	Eggplant	3 × 10^0^	6 × 10^0^	-	[25]
			2022	Bangladesh	Eggplant	-	3 × 10^1^	-	[26]
3.	Erebidae	*Laspeyresia caryana*	1970–1972	Georgia	Pecan	3 × 10^0^	2 × 10^1^	7 × 10^2^	[27]
4.1.1	Gelechiidae	*Pectinophora gossypiella*	1954	Texas	Cotton	4 × 10^0^	2 × 10^1^	3 × 10^5^	[28]
4.1.2			2020	India	Cotton	-	1 × 10^0^	6 × 10^3^	[29]
4.2		*Phthorimaea operculella*	2019–2020	Turkey	Potato	1 × 10^−2^	4 × 10^0^	3 × 10^3^	[30]
4.3.1		*Tuta absoluta*	2013	Iran	Tomato	1 × 10^0^	2 × 10^1^	9 × 10^3^	[31]
4.3.2			2019–2020	Turkey	Tomato	4 × 10^−1^	8 × 10^0^	5 × 10^3^	[32]
			2021	Brazil	Tomato	1 × 10^0^	3 × 10^0^	7 × 10^3^	[33]
4.3.3			2022	Pakistan	Tomato	4 × 10^−1^	8 × 10^0^	5 × 10^3^	[34]
	Geometridae	*Ectropis obliqua*	2014	China	Tea	-	9 × 10^0^	1 × 10^2^	[35]
5.1		*Glena bipennaria*	2014	Brazil	Eucalyptus	4 × 10^2^	1 × 10^1^	-	[36]
5.2		*Thyrinteina arnobia*	2015	Brazil	Eucalyptus	4 × 10^2^	1 × 10^1^	2 × 10^6^	[36]
6.	Limacodidae	*Macroplectra nararia*	2009	India	Coconut	3 × 10^0^	5 × 10^1^	1 × 10^4^	[37]
7.	Lymantriidae	*Ocnerogyla amanda*	2021	Iraq	Ficus	5 × 10^0^	2 × 10^1^	5 × 10^3^	[38]
8.1	Noctuidae	*Autographa californica*	1967–1969	Arizona	Lettuce	1 × 10^4^	5 × 10^2^	1 × 10^4^	[39]
8.2		*Estigmene acrea*	1967–1969	Arizona	Lettuce	1 × 10^4^	5 × 10^2^	3 × 10^5^	[39]
8.3		*Feltia subterranean*	1967–1969	Arizona	Lettuce	1 × 10^4^	5 × 10^2^	7 × 10^4^	[39]
8.4.1		*Helicoverpa armigera*	2009–2010	Pakistan	Chickpea	2 × 10^1^	1 × 10^1^	4 × 10^3^	[40]
8.4.2			2017–2018	Pakistan	Mungbean	1 × 10^0^	2 × 10^0^	1 × 10^3^	[41]
8.4.3			2014–2019	China	Cotton	5 × 10^0^	4 × 10^1^	1 × 10^4^	[42]
			2019	India	Groundnut	1 × 10^0^	2 × 10^1^	4 × 10^3^	[43]
8.4.6									
8.5.1		*Heliothis virescens*	1965–1966	Florida	Tobacco	1 × 10^1^	2 × 10^1^	-	[44]
8.5.3			1966–1968	Florida	Tobacco	1 × 10^4^	1 × 10^1^	3 × 10^2^	[45]
8.5.4			1967	Texas	Cotton	2 × 10^1^	6 × 10^0^	1 × 10^3^	[46]
8.5.5			1968–1969	Texas	Grass	0	0	0	[47]
8.5.6			1970–1971	Texas	Cot/Corn/Sor	1 × 10^3^	2 × 10^1^	3 × 10^3^	[48]
8.6.1		*Heliothis zea*	1936	California	Tomato	-	-	+	[49]
8.6.2			1958–1962	Indiana	Corn	1 × 10^0^	1 × 10^1^	-	[21]
			1965–1966	North Carolina	Tobacco	-	5 × 10^1^	5 × 10^4^	[50]
			1967	North Carolina	Tobacco	-	1 × 10^1^	7 × 10^3^	[51]
8.6.3			1966–1967	Mexico	Corn	2 × 10^1^	8 × 10^1^	4 × 10^4^	[52]
8.6.4			1966–1970	South Carolina	Cotton	1 × 10^0^	3 × 10^1^	5 × 10^3^	[53]
8.6.5			1966–1968	Florida	Tobacco	1 × 10^4^	1 × 10^1^	2 × 10^3^	[45]
8.6.8			1967	Texas	Cotton	2 × 10^1^	6 × 10^0^	2 × 10^3^	[46]
8.6.9			1967–1969	Arizona	Lettuce	1 × 10^4^	5 × 10^2^	1 × 10^6^	[39]
8.6.10			1970–1971	Texas	Cot/Cor/Sor	1 × 10^3^	2 × 10^1^	2 × 10^4^	[48]
8.6.13			1975	Texas	Cot/Cor/Sor	-	1 × 10^1^	1 × 10^4^	[54]
		*Pseudaletia unipunctata*	1967	North Carolina	Tobacco	-	1 × 10^1^	4 × 10^3^	[51]
8.7.1		*Spodoptera exigua*	1967–1969	Arizona	Lettuce	1 × 10^4^	5 × 10^2^	3 × 10^6^	[39]
8.7.2			1983–1990	South Africa	Sugarcane	-	7 × 10^0^	1 × 10^2^	[55]
8.7.3			2018	Indonesia	Onion	5 × 10^−2^	9 × 10^0^	3 × 10^3^	[56]
		*Spodoptera frugiperda*	2018–2019	Ethiopia	Corn	2 × 10^−2^	4 × 10^1^	2 × 10^3^	[57]
8.8.1		*Spodoptera litura*	2018	Indonesia	Cabbage	1 × 10^−2^	4 × 10^1^	2 × 10^3^	[24]
8.8.2			2017–2018	Pakistan	Mungbean	1 × 10^0^	2 × 10^0^	2 × 10^3^	[41]
			2019	India	Groundnut	1 × 10^0^	2 × 10^1^	8 × 10^3^	[43]
8.9		*Spodoptera ornithogalli*	1967–1969	Arizona	Lettuce	1 × 10^4^	5 × 10^2^	1 × 10^5^	[39]
8.10.1		*Trichoplusia ni*	1965	Florida	Tobacco	1 × 10^1^	2 × 10^1^	-	[44]
8.10.3			1967	Texas	Cotton	2 × 10^1^	6 × 10^0^	1 × 10^4^	[46]
8.10.4			1967–1969	Arizona	Lettuce	1 × 10^4^	5 × 10^2^	3 × 10^6^	[39]
9.	Plutellidae	*Plutella xylostella*	2018	Indonesia	Cabbage	1 × 10^−2^	6 × 10^0^	5 × 10^2^	[24]
10.	Pterophoridae	*Platyptilia carduitactyla*	1933	California	Artichoke	2 × 10^0^	4 × 10^0^	6 × 10^2^	[58]
11.1.1	Sphingidae	*Manduca sexta*	1963	North Carolina	Tobacco	6 × 10^4^	7 × 10^2^	4 × 10^4^	[59]
11.1.4			1954–1956	Virginia	Tobacco	6 × 10^0^	9 × 10^0^	7 × 10^3^	[60]
11.1.5			1964–1966	South Carolina	Tobacco	3 × 10^4^	3 × 10^2^	7 × 10^4^	[46]
			1965–1966	North Carolina	Tobacco	-	6 × 10^1^	5 × 10^3^	[50]
			1967	North Carolina	Tobacco	-	1 × 10^1^	5 × 10^3^	[51]
11.2.1		*M. quinquemaculata*	1963–1964	North Carolina	Tobacco	5 × 10^4^	7 × 10^2^	5 × 10^4^	[59]
11.2.3			1954–1956	Virginia	Tobacco	6 × 10^0^	9 × 10^0^	1 × 10^4^	[60]
			1965–1966	North Carolina	Tobacco	-	6 × 10^1^	1 × 10^3^	[50]
11.3.1		*Ms*, *Mq*	1942	Maryland	Tobacco	3 × 10^2^	7 × 10^1^	1 × 10^4^	[61]
11.3.2			1958–1962	Indiana	Tomato	1 × 10^0^	1 × 10^1^	-	[21]
11.3.3			1965–1966	Kentucky	Tobacco	3 × 10^4^	3 × 10^2^	2 × 10^6^	[62]
12.1	Tortricidae	*Archips argyrospila*	1929	New York	Apple	2 × 10^0^	1 × 10^2^	2 × 10^4^	[63]
12.2.1		*Cydia pommonella*	1933	California	Apple	-	4 × 10^0^	3 × 10^3^	[64]
			1933–1936	New York	Apple	-	-	-	[65]
			1934–1936	Indiana	Apple	2 × 10^0^	4 × 10^1^	3 × 10^2^	[66]
			2020–2021	Turkey	Apple	9 × 10^0^	1 × 10^1^	5 × 10^3^	[67]
		*Eucosma ocellana*	1929	New York	Apple	2 × 10^0^	1 × 10^2^	3 × 10^4^	[63]
13.	Xyloryctidae	*Opisina arenosella*	2004	India	Coconut	7 × 10^1^	2 × 10^1^	4 × 10^2^	[68]

**Table 2:** Parameters related to efficiency of *LT_mt_*. (1) Trap attributes: ratio of target pests versus beneficial insects (*n_p_*/*n_b_*); height (*h_i_*) of *LTs* above ground. Demography of target pests: sex ratios of adults in *LTs* (*n*_♀_/*n*_♂_).

Year	Species	Trap Attributes		Demography of Pests		Crop Damage	Refs.
		*n_p_/n_b_*	h_i_	*n_♀_*/*n*_♂_	♀_(egg)_		
1929	*Aa*, *Eo*	Yes	Yes	Yes	Yes	Yes	[63]
1933	*Cp*		Yes	Yes		Yes	[64]
1933–1936	*Cp*					Yes	[65]
1934–1936	*Cp*					Yes	[66]
1936	*Hz*	Yes		Yes		Yes	[49]
1938	*On*		Yes				[20]
1942	*Mq*, *Ms*			Yes		Yes	[61]
1954	*Pg*		Yes	Yes			[28]
1954–1956	*Mq*, *Ms*			Yes		Yes	[60]
1958–1959	*Mq*, *Ms*					Yes	[62]
1958–1962	*Hz*, *Ms*, *Mq*, *On*					Yes	[21]
1963–1964	*Mq*, *Ms*			Yes		Yes	[59]
1964–1966	*Ms*			Yes	Yes	Yes	[62]
1965–1966	*Hz*, *Ms*, *Mq*			Yes			[50]
1965–1966	*Hv*, *Tn*			Yes		Yes	[44]
1965–1966	*Mq*, *Ms*			Yes		Yes	[63]
1966–1967	*Hz*			Yes	Yes	Yes	[52]
1966–1968	*Hv*, *Hz*			Yes	Yes		[45]
1966–1970	*Hz*	Yes				Yes	[53]
1967	*Hv*, *Hz*, *Tn*			Yes	Yes		[46]
1967	*Hz*, *Ms*, *Pu*		Yes				[51]
1967–1969	*Ac*, *Ea*, *Fs*, *Hz*, *Se*, *So*			Yes	Yes	Yes	[39]
1968–1969	*Hv*			Yes			[47]
1970	*Ds*			Yes	Yes		[22]
1970–1971	*Hv*, *Hz*					Yes	[48]
1970–1972	*Lc*					Yes	[27]
1975	*Hz*			Yes			[54]
1983–1990	*Se*	Yes					[55]
1993	*Ci*	Yes		Yes			[23]
2003–2005	*Zp*					Yes	[4]
2004	*Oa*		Yes				[68]
2009	*Mn*		Yes	Yes			[37]
2009–2010	*Ha*		Yes			Yes	[40]
2013	*Ta*	Yes				Yes	[31]
2014	*Eo*	Yes					[35]
2014–2015	*Zp*						[19]
2017–2018	*Ha*, *Sl*	Yes		Yes		Yes	[41]
2018	*Se*					Yes	[56]
2018	*Lo*					Yes	[25]
2018	*Cb, Px*, *Sl*					Yes	[24]
2018–2019	*Sf*					Yes	[57]
2019–2020	*Ta*	Yes		Yes		Yes	[32]
2019–2020	*Po*			Yes		Yes	[30]
2019–2020	*Ha*	Yes				Yes	[43]
2020	*Pg*	Yes					[29]
2020–2021	*Cp*	Yes		Yes		Yes	[67]
2022	*Ta*			Yes		Yes	[34]
2022	*Lo*					Yes	[26]

*Aa*: *Archips argyrospila*; *Ac*: *Autographa californica*; *Cb*: *Crocidolomia binotalis*; *Ci*: *Coniesta ignefusalis*; *Cp*: *Cydia pommonella*; *Ds*: *Diatraea saccharalis*; *Ea*: *Estigmene acrea*; *Eo*: *Ecosma ocellana*; *Fs*: *Felta subterranean*; *Ha*: *Heliothis armigera*; *Hv*: *Heliothis virescens*; *Hz*: *Heliothis zea*; *Lc*: *Laspeyresia caryana*; *Lo*: *Leucinodes orbonalis*; *Mn*: *Macroplectra nararia*; *Mq*: *Manduca quinquemaculata*; *Ms*: *Manduca sexta*; *Oa*: *Opsina arenosella*; *On*: *Ostria nubilalis*; *Pg*: *Pectinophora gossypiella*; *Pu*: *Pseudaletia unipunctata*; *Px*: *Plutella xylostella*; *Se*: *Spodoptera exigua*; *Sf*: *Spodoptera frugiperda*; *Sl*: *Spodoptera litura*; *So*: *Sprodoptera ornithogalli*; *Ta*: *Tuta absoluta*; *Tn*: *Trichoplusia ni*; *Zp*: *Zeuzera pyrina*.

**Table 3:** Parameters used to model efficacy of light trap-based mass trapping.

Abundance of Pests		
*n_p_*	(*n*_♂_, *n*_♀_)	Number of pests per trap during flight season
*n_b_*	*←* *p*	Number of beneficial insects (non-pests) per trap
Ʃ*n_p_*	(*n*_♂_, *n*_♀_)	Cumulative number of pests in all traps
*N_p_*	(*n*_♂_, *n*_♀_)	Number of resident pests available for trapping
*w_p_*	(*w*_♂_, *w*_♀_)	Weight of pests per trap during flight season
*w_b_*	*←* *p*	Weight of beneficial insects (non-pests) per trap
Ʃ*w_p_*	(*w*_♂_, *w*_♀_)	Cumulative weight of pests in all traps
*S*		Impact of ♂ versus ♀ in light traps on crop damage
*F*		Average fecundity of females for any given pest
Attributes of *LTs*		
*d_i/_d_ij_*		Overall trap design optimizing (*n_p_*/*n_b_*) or (*w_p_*/*w_b_*)
	*l_i_*–*l_ij_*	Optimal light source {wavelength, intensity, surface, etc.}
	*f_i_*–*f_ij_*	Optimal funnel design
	*v_i_*–*v_ij_*	Optimal volume of receptacle
*h_i_*		Height of trap, above ground
*T*		Number of traps, per ha
*r*		Range of attraction of trap, in *m*
Behavioral responses of adult pests to *LTs*		
*k*		Logistic response of adults in relation to density of traps (*T*)
*Ω*		Proportion of resident adults captured in traps (Ʃ*w_p_*/*N_p_*)
*c*		Constant proportion of adults captured in *LTs*, i.e., *Ω _┴_ N_p_*
*e*		Exponential proportion of adults captured in *LTs*, i.e., *Ω* ^┬^ *N_p_*
*L*		Proportion of eggs laid by females prior to capture in traps

**Table 4:** Demographic parameters of adult spruce budworms captured in light traps deployed in west coast of Newfoundland in 2019 (Figure 1). One trap was deployed inland in Pasadena (PAS), 40 km from coast. Other 15 traps were deployed within 3 km from coast, either within Gros Morne National Park [Rocky Harbor (RH), Sally’s Cove (SC), and Cowhead (CH) or north of Gros Morne [Three Mile Road (TMR), Arches (ARC), Portland Creek (PC), and Daniel Harbor (DH)] (Figure 1). Average date of capture is expressed as number of days after June 30th. Average parameter values (Table 3) are as follows on per trap basis. I. Abundance: number and fresh biomass of budworms (*n_p_* and *w_p_*), weight of bycatch (*w_b_*: all non-budworms in light traps). II. Seasonality: average date of capture for *n*_♂_, *n*_♀_. III. Sex ratio {*n*_♀_/(*n*_♂_ + *n_♀_*)}. IV. Protandry: average date of flight of ♀—average date of flight of ♂.

Location	Site	Interval	Abundance			Mean Capture Day		P (♀)	Protandry
			*n_p_* (1000)	*w_p_* (kg)	*w_b_* (kg)	*n*_♂_	*n*_♀_		
Inland	PAS	25–46	13.5	0.20	0.50	33.5	34.1	0.133	0.6
Park	RH–1	17–55	29.3	0.38	0.31	34.1	36.3	0.201	2.2
	RH–2		23.3	0.32	0.25	34.5	36.2	0.321	1.7
	RH–3	36.0	0.48	0.30	33.9	35.7	0.158	1.8	
	RH–4	44.9	0.63	0.37	34.6	35.5	0.255	0.9	
	SC–1	18–57	96.8	1.45	0.48	36.1	39.3	0.222	3.2
	SC–2		79.7	1.21	0.55	36.9	39.7	0.247	2.8
	SC–3		96.9	1.53	0.90	36.0	38.3	0.205	2.3
	SC–4		121.8	1.87	0.93	36.0	38.4	0.208	2.4
	CH–1	21–60	71.6	1.04	0.93	35.7	38.8	0.225	3.1
	CH–2		38.8	0.55	0.38	34.6	36.7	0.219	2.1
North	TMR	25–59	92.6	1.49	0.70	37.3	39.1	0.175	1.8
	ARC	25–60	76.0	1.20	0.50	39.2	41.7	0.244	2.5
	PC	31–58	53.7	0.81	0.46	37.0	38.7	0.218	1.7
	DH–1	32–60	18.3	0.25	0.18	38.8	37.3	0.240	−1.5
	DH–2		60.2	0.90	0.53	42.9	38.4	0.250	4.5

**Table 5:** Numerical (*n*) and biomass (*w*) abundance of male and female spruce budworms captured in light traps deployed along west coast of Newfoundland in 2019, including sex ratio assessments [♀/(♀ + ♂)]. Index *w*_♀_/*n*_♀_ for each trap corresponds to [♀/(♀ + ♂)*_w_*]/[♀/(♀ + ♂)*_n_*].

Location	Site	*n_p_* (1000)			*w_p_* (100 g)			*w*_♀_/*n*_♀_
		♀	♂	♀/(♀ + ♂)	♀	♂	♀/(♀ + ♂)	
Inland	PAS	1.7	16.8	0.133	37.4	159.1	0.190	1.43
Park	RH–1	5.9	23.1	0.204	105.1	275.1	0.276	1.35
	RH–2	6.3	15.5	0.321	104.5	186.7	0.359	1.12
	RH–3	5.7	30.3	0.158	105.5	0.219	377.3	1.38
	RH–4	11.4	33.4	0.255	200.4	428.6	0.319	1.25
	SC–1	21.1	75.0	0.222	384.2	1057.5	0.266	1.20
	SC–2	19.5	59.9	0.247	335.1	867.9	0.279	1.13
	SC–3	18.5	76.9	0.205	361.3	1142.7	0.240	1.17
	SC–4	23.5	93.9	0.208	459.6	1342.0	0.255	1.23
	CH–1	14.2	48.4	0.225	246.2	676.2	0.267	1.19
	CH–2	8.5	30.2	0.220	152.6	401.4	0.275	1.25
North	TMR	14.4	76.2	0.175	271.7	1171.2	0.188	1.07
	ARC	16.8	56.4	0.244	330.5	825.9	0.286	1.17
	PC	11.7	42.0	0.218	216.0	589.6	0.268	1.23
	DH–1	3.8	14.2	0.207	66.1	180.7	0.268	1.29
	DH–2	14.3	44.4	0.250	269.3	604.8	0.308	1.23

## Supplementary Materials

The following supporting information can be downloaded at: https://www.mdpi.com/article/10.3390/insects15040267/s1, ANNEX I: Light trapping protocols.
