# Supplementary material for: Mass Trapping Lepidopteran Pests with Light Traps, with Focus on Tortricid Forest Pests: What If?
Source: Insects. 2024 Apr 12;15(4):267. doi: 10.3390/insects15040267 (PMC11049961; doi:10.3390/insects15040267)
Supplement: Supplementary file 1 [file insects-15-00267-s001.zip › insects-2925112-supplementary.pdf]

ANNEX I

Sixteeen stainless steel vane light traps (*LT*) (Leptraps, Georgetown, KY, USA) with a 15-W white neon tube as light source and powered by marine batteries were used to capture adult budworms in forest stands dominated by balsam fir at eight locations in central western Newfoundland in 2019 (Fig. 1). Traps were suspended on a rope 3–4 m above ground between the trunks of two firs. Each trap was wired to a 12-V marine battery for power, and batteries were replaced every 4–6 days to prevent discharge. Insects captured at *LT* were killed with insecticide strips (Vaportape II; Hercon Environmental, Emigsville, PA, USA).

The rational for collecting *LT* samples every day was fourfold. (1) Daily records of abundance are key to recognize immigration at *LT*, in particular post-migratory longevity of adults [2]. (2) Maintain high quality of biological material to be processed: insects kept at light traps for intervals as short as two days rapidly deteriorate, as indicated by captures of hundreds burying beetles (Nicrophonidae, Silphidae) attracted to decomposition volatiles. (3) Limit exposure to wing scales / bycatch allergens. (4) Reduce incidence of light trap malfunction through daily inspection of battery power charge.

During collection, insects were transferred from *LT* buckets into plastic boxes then covered with a lid and labelled by day and site; lids with a screen middle portion were used during rainy days to prevent decomposition of wet specimens. The transfer process lasted less than 30 seconds per sample. All trap samples were collected and brought to the laboratory at Pasadena on the same day. Within thirty minutes after *LT* samples arrived at Pasadena, they were spread on white cardboards placed on the floor of the laboratory (drying process). The laboratory consisted of a large room (6 x 8 m) with ten screened windows kept open to ensure good ventilation and reduce exposure to *LT* allergens. Samples were processed in the laboratory within 48 hr after collection.

In a first step, the fresh weight of light trap samples was recorded to nearest 0.5 g, and budworm specimens were then separated from the bycatch using one of two procedures. (1) When density of budworms was low (< 250 adults / trap / night; interval between 17 – 30 July and 24 – 29 August), all budworm specimens were separated from the bycatch. (2) During periods of high budworm abundance (31 July to 23 August), budworm specimens were separated from *LT* subsamples of known fresh weight; each subsample included a minimum of 250 budworms. For each trap and day, fresh weight of male and female budworm samples (each with known numbers of individuals), as well as the corresponding bycatch, were evaluated to the nearest 0.001 g.

Two distinct steps were used to process light trap samples before 2019: budworms were first separated from the bycatch, and the sex of each specimen determined under a stereomicroscope. Processing time was greatly reduced in 2019 by combining the separation and sexing of budworms as a one-step process, first using magnification glasses in early season, then after a 2 wk 'practice period' with naked eyes.

Summary of results obtained at 16 light traps deployed on west coast of Newfoundland in 2019 (Table 3). Number of light traps samples (*N*) corresponding to different number of spruce budworms are provided on daily basis either in terms of numerical (n_p) or biomass abundance (w_p).

n_p / day	N	w_p / day (g)	N

500 –1000	46	5 –10	50
1000 – 2000	57	11 – 20	55
2000 –4000	41	21 – 40	37
4000 –8000	53	41 – 80	46
8000 – 16 000	30	81 – 160	47
> 16 000	3	> 160	17
